# *catena*-Poly[neodymium(III)-bis­[μ-*N*-(dimorpholinophosphor­yl)benzene­sulfonamidato]-sodium(I)-bis­[μ-*N*-(dimorpholinophosphor­yl)benzene­sulfonamidato]]

**DOI:** 10.1107/S1600536810008214

**Published:** 2010-03-13

**Authors:** Iuliia O. Shatrava, Tatyana Yu. Sliva, Vladimir A. Ovchynnikov, Irina S. Konovalova, Vladimir M. Amirkhanov

**Affiliations:** a National Taras Shevchenko University, Department of Chemistry, Volodymyrska str. 64, 01033 Kyiv, Ukraine; b STC "Institute for Syngle Crystals", 60 Lenina ave., Khar’kov 61001, Ukraine

## Abstract

The cubic crystal structure of the title compound, [NaNd(C_14_H_21_N_3_O_5_PS)_4_]_*n*_, is composed of one-dimensional polymeric chains propagating in [100], built up from [Nd(C_14_H_21_N_3_O_5_PS)_4_]^−^ anions and sodium cations functioning as linkers. In the complex anion, the Nd^3+^ ion has an eightfold coordination environment formed by the sulfonyl and phosphoryl O atoms of four bidentate chelating *N*-(dimorpholinophosphor­yl)benzene­sulfonamidate ligands: the resulting NdO_8_ polyhedron can be described as inter­mediate between dodeca­hedral and square anti­prismatic. The sodium ion adopts an NaO_4_ tetra­hedral geometry arising from four monodentate benzene­sulfonamidate ligands. The resulting crystal structure is unusual because it contains substantial voids (800 Å^3^ per unit cell), within which there is no evidence of included solvent.

## Related literature

For general background to the use of bidentate ligands in ring closure in coordination compounds, see: Casas *et al.* (1995 ▶); Amirkhanov *et al.* (1997 ▶); Ly & Woollins (1998 ▶). For applications of the chelates formed, see: Zazybin *et al.* (2006 ▶); Karande *et al.* (2003 ▶); Morgalyuk *et al.* (2005 ▶); Xu & Angell (2000 ▶). For lanthanide compounds of general formula Na[Ln(*L* ^1^)_4_]_*n*_ where H*L* ^1^ is C_6_H_5_S(O)_2_NHPO(OCH_3_)_2_, see: Moroz *et al.* (2007 ▶). For the synthesis of the ligand, see: Kirsanov & Shevchenko (1954 ▶); Oyamada & Morimura (1960 ▶). For inter­pretation of coordination polyhedra, see: Porai-Koshits & Aslanov (1972 ▶). For bond lengths in similar compounds, see: Sokolov *et al.* (2007 ▶); Sokolnicki *et al.* (1998 ▶).
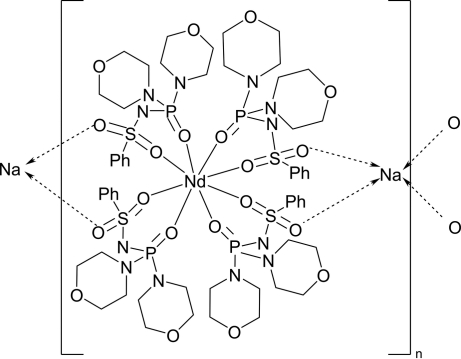


## Experimental

### 

#### Crystal data

[NaNd(C_14_H_21_N_3_O_5_PS)_4_]
*M* *_r_* = 1664.72
Cubic, 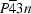

*a* = 22.943 (5) Å
*V* = 12077 (5) Å^3^
*Z* = 6
Mo *K*α radiation
μ = 0.90 mm^−1^
*T* = 293 K
0.60 × 0.40 × 0.30 mm

#### Data collection

Oxford Diffraction KM-4 Xcalibur diffractometer with a Sapphire3 detector
Absorption correction: multi-scan (*CrysAlis RED*; Oxford Diffraction, 2006 ▶) *T*_min_ = 0.614, *T*_max_ = 0.774
66051 measured reflections
5883 independent reflections
3713 reflections with *I* > 2σ(*I*)
*R*_int_ = 0.113

#### Refinement

*R*[*F* ^2^ > 2σ(*F* ^2^)] = 0.095
*wR*(*F* ^2^) = 0.178
*S* = 1.42
5883 reflections
174 parameters
1 restraint
H-atom parameters constrained
Δρ_max_ = 1.17 e Å^−3^
Δρ_min_ = −0.75 e Å^−3^
Absolute structure: Flack (1983 ▶), 2727 Friedel pairs
Flack parameter: 0.05 (3)

### 

Data collection: *CrysAlis CCD* (Oxford Diffraction, 2006 ▶); cell refinement: *CrysAlis RED* (Oxford Diffraction, 2006 ▶); data reduction: *CrysAlis RED*; program(s) used to solve structure: *SHELXS97* (Sheldrick, 2008 ▶); program(s) used to refine structure: *SHELXL97* (Sheldrick, 2008 ▶); molecular graphics: *ORTEP-3* (Farrugia, 1997 ▶); software used to prepare material for publication: *WinGX* (Farrugia, 1999 ▶).

## Supplementary Material

Crystal structure: contains datablocks I, global. DOI: 10.1107/S1600536810008214/hb5339sup1.cif

Structure factors: contains datablocks I. DOI: 10.1107/S1600536810008214/hb5339Isup2.hkl

Additional supplementary materials:  crystallographic information; 3D view; checkCIF report

## Figures and Tables

**Table 1:** Selected bond lengths (Å)

Nd1—O1	2.376 (4)
Nd1—O2	2.532 (4)
Na1—O3	2.282 (4)

**Fig. 1.**
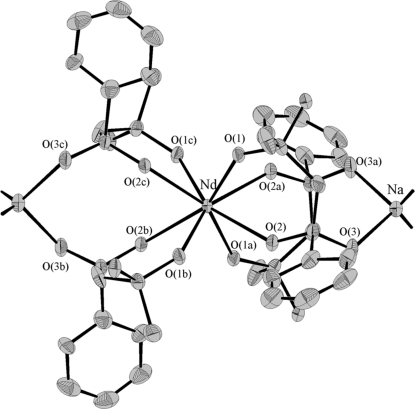
A view of Na[Nd(L)4]n with displacement ellipsoids shown at the 30% probability level. H atoms and morpholine rings have been omitted for clarity.

**Fig. 2.**
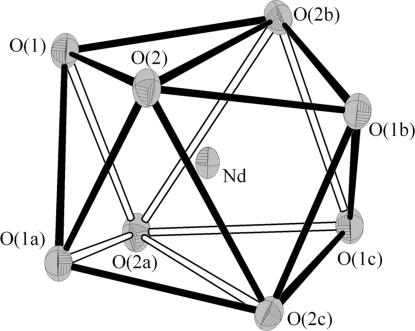
Polyhedror of Nd3+ in (I).

**Fig. 3.**
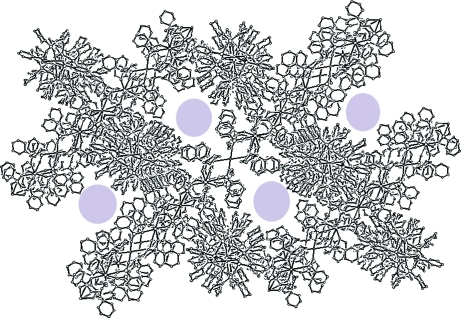
Motif of packing of (I) viewed along z (all H atoms are omitted for clarity).

## supplementary crystallographic information

### Comment

Lots of bidentate ligands under coordination form closure rings through the donor atoms binding to the same metal. The most used ligands are those derived from that containing oxygen, nitrogen, phosphorus and sulphur atoms (Casas *et al.*, 1995; Amirkhanov *et al.*, 1997; Ly *et al.*, 1998). Such chelates may be used in catalysis (Zazybin *et al.*, 2006), metal extraction (Karande *et al.*, 2003; Morgalyuk *et al.*, 2005), bioinorganic chemistry (Xu *et al.*, 2000). Phosphorylated sulphonylamides of a general view RS(O)_2_NHP(O)(NR_2_)_2_ could be applied for obtaining of lanthanide coordination compounds and presence of sulfono-group oxygen atom as addititious coordination centre gives a challenging opportunity to use them as convenient building blocks for syntheses of bi- and poly-nuclear compounds.

The results of lanthanide compounds investigation with one of the phosphorylated sulphonylamides representative – C_6_H_5_S(O)_2_NHPO(OCH_3_)_2_ (HL^1^) of general formula Na[Ln(L^1^)_4_]_n_ were already reported (Moroz *et al.*, 2007).

We now report the synthesis and investigation of tetrakis - complex of the composition {Na[Nd(L)_4_]}_n_, (I) (Fig.1), where L^-^ is dimorpholinephenylsulphonylamidophosphate (C_6_H_5_S(O)_2_NPO(NC_4_H_8_O)_2_)^-^. The synthesis of HL was carried out according to (Oyamada *et al.*, 1960; Kirsanov *et al.*, 1954), using benzenesulfonamide and morpholine.

The molecular structure of title compound contains 1D polymer chain, formed by [Nd(L)_4_)]^-^ anion and sodium cation as a linker. In complex anions the neodymium atoms have 8-fold coordination environment formed by oxygen atoms of SO_2_ and PO groups of four bidentate chelate ligands (Fig. 2). According to Porai-Koshits (Porai-Koshits & Aslanov, 1972) the resulting coordination polyhedra can be interpreted as a medium conformation between dodecahedron (δ_1_ = δ_2_ = δ_3_ = δ_4_ = 29.5 °) and square antiprism (δ_1_ = δ_2_ = 0 °; δ_3_ = δ_4_ = 52.5 °) for Nd atom (interplanar angles in polyhedra for Nd δ_1_ = δ_2_ = 26.2 °; δ_3_ = δ_4_ = 51.8 °).

The Nd – O(P) bond lengths (2.376 (4) Å) are shorter than Nd – O(S) (2.532 (4) Å) that can be explained by higher affinity of phosphoryl group to lanthanide ions. The P – O (1.499 (4) Å), N(1) – P (1.613 (6) Å) bond lengths are also comparable with the values observed for similar compounds (Sokolnicki *et al.*, 1998; Sokolov *et al.*, 2007). The average P—N (morpholine substituents) distance (1.628 (7) Å) is larger than P—N(1) bond length in chelate core because of the conjugation in S(O)_2_NP(O) fragment. The metallocycles are almost flat with a deviation of the N(1) atom from the mean plane defined by the six atoms NdO(1)P(1) N(1)S(1)O(2) of 0.24496 Å.

The bonding of complex anions in polymer structure is provided by Na ions. The Na polyhedron is a distorted tetrahedron, formed by two SO oxygens from different anions.

The crystal structure is unusual: it contains substantial voids (800 Å^3^) within which there is no evidence for included solvent (Fig. 3). The crystals remained glass-clear being on air.

### Experimental

Nd(NO_3_)_3_7H_2_O (0.087 g, 1 mmol) was dissolved in 10 ml of i-PrOH and added to 10 ml of a solution of NaL (0.3 g, 4 mmol) in a mixture of methanol and i-PrOH (1:1). After 30 min the precipitate of NaNO_3_ was filtered off. The resulting clear solution was left for crystallization in a vacuum desiccator. The resulting light violet blocks of (I) were separated by filtration after 48 h, washed with cool i-PrOH (5 ml) and finally dried in air. Yield: 85–90%. IR (KBr pellet, cm^-1^): 1240, 1030 (s, SO_2_) and 1140 (s, PO).

### Refinement

All hydrogen atoms were located from electron density difference maps and included in the refinement in the riding motion approximation with U_iso_ constrained to be 1.5 times U_eq_ of the carrier atom for the methyl groups and 1.2 times U_eq_ of the carrier atom for the other atoms.

### Crystal data

**Table:**

[NaNd(C_14_H_21_N_3_O_5_PS)_4_]	Mo *K*α radiation, λ = 0.71069 Å
*M**_r_* = 1664.72	Cell parameters from 68542 reflections
Cubic, *P*43*n*	θ = 2.8–32.1°
*a* = 22.943 (5) Å	µ = 0.90 mm^−^^1^
*V* = 12077 (5) Å^3^	*T* = 293 K
*Z* = 6	Block, light violet
*F*(000) = 5154	0.60 × 0.40 × 0.30 mm
*D*_x_ = 1.373 Mg m^−^^3^	

### Data collection

**Table:**

Oxford Diffraction KM-4 Xcalibur diffractometer with a Sapphire3 detector	5883 independent reflections
Radiation source: fine-focus sealed tube	3713 reflections with *I* > 2σ(*I*)
graphite	*R*_int_ = 0.113
Detector resolution: 16.1827 pixels mm^-1^	θ_max_ = 30.0°, θ_min_ = 2.8°
ω scans	*h* = −31→32
Absorption correction: multi-scan (*CrysAlis RED*; Oxford Diffraction, 2006)	*k* = −30→32
*T*_min_ = 0.614, *T*_max_ = 0.774	*l* = −32→32
66051 measured reflections	

### Refinement

**Table:**

Refinement on *F*^2^	Secondary atom site location: difference Fourier map
Least-squares matrix: full	Hydrogen site location: inferred from neighbouring sites
*R*[*F*^2^ > 2σ(*F*^2^)] = 0.095	H-atom parameters constrained
*wR*(*F*^2^) = 0.178	*w* = 1/[σ^2^(*F*_o_^2^) + (0.0311*P*)^2^ + 27.6297*P*] where *P* = (*F*_o_^2^ + 2*F*_c_^2^)/3
*S* = 1.42	(Δ/σ)_max_ = 0.053
5883 reflections	Δρ_max_ = 1.17 e Å^−^^3^
174 parameters	Δρ_min_ = −0.74 e Å^−^^3^
1 restraint	Absolute structure: Flack (1983), 2727 Friedel pairs
Primary atom site location: structure-invariant direct methods	Flack parameter: 0.05 (3)

### Special details

**Table:**

Experimental. CrysAlis RED, (Oxford Diffraction Ltd., 2007) Empirical absorption correction using spherical harmonics, implemented in SCALE3 ABSPACK scaling algorithm.
Geometry. All esds (except the esd in the dihedral angle between two l.s. planes) are estimated using the full covariance matrix. The cell esds are taken into account individually in the estimation of esds in distances, angles and torsion angles; correlations between esds in cell parameters are only used when they are defined by crystal symmetry. An approximate (isotropic) treatment of cell esds is used for estimating esds involving l.s. planes.
Refinement. Refinement of *F*^2^ against ALL reflections. The weighted *R*-factor wR and goodness of fit *S* are based on *F*^2^, conventional *R*-factors *R* are based on *F*, with *F* set to zero for negative *F*^2^. The threshold expression of *F*^2^ > σ(*F*^2^) is used only for calculating *R*-factors(gt) etc. and is not relevant to the choice of reflections for refinement. *R*-factors based on *F*^2^ are statistically about twice as large as those based on *F*, and *R*- factors based on ALL data will be even larger.

### Fractional atomic coordinates and isotropic or equivalent isotropic displacement parameters (Å^2^)

**Table:**

	*x*	*y*	*z*	*U*_iso_*/*U*_eq_	
Nd1	0.2500	0.0000	0.5000	0.03890 (11)	
Na1	0.5000	0.0000	0.5000	0.0591 (11)	
P1	0.34264 (7)	−0.11150 (8)	0.56551 (8)	0.0580 (4)	
S1	0.38783 (6)	−0.07841 (7)	0.45680 (7)	0.0542 (4)	
C1	0.3831 (3)	−0.1414 (3)	0.4113 (3)	0.0561 (17)	
N1	0.3939 (2)	−0.1001 (3)	0.5188 (2)	0.0784 (19)	
N2	0.3754 (2)	−0.1023 (3)	0.6282 (3)	0.0907 (14)	
N3	0.3220 (3)	−0.1790 (3)	0.5648 (4)	0.126 (2)	
O2	0.33520 (14)	−0.04482 (17)	0.44606 (16)	0.0497 (10)	
O3	0.44068 (16)	−0.0500 (2)	0.4378 (2)	0.0719 (13)	
O1	0.28838 (15)	−0.07562 (17)	0.55946 (17)	0.0526 (11)	
O4	0.4251 (3)	−0.0792 (4)	0.7367 (2)	0.134 (2)	
O5	0.2927 (4)	−0.2972 (3)	0.5510 (4)	0.163 (3)	
C2	0.4310 (3)	−0.1778 (3)	0.4091 (3)	0.076 (2)	
H2A	0.4651	−0.1699	0.4296	0.092*	
C3	0.4246 (4)	−0.2277 (3)	0.3737 (4)	0.108 (3)	
H3A	0.4562	−0.2528	0.3701	0.129*	
C4	0.3754 (5)	−0.2411 (4)	0.3447 (4)	0.112 (3)	
H4A	0.3734	−0.2741	0.3212	0.135*	
C5	0.3306 (5)	−0.2067 (4)	0.3505 (4)	0.111 (3)	
H5A	0.2960	−0.2167	0.3321	0.133*	
C6	0.3325 (4)	−0.1552 (3)	0.3833 (3)	0.079 (2)	
H6A	0.3000	−0.1311	0.3859	0.095*	
C7	0.4355 (3)	−0.0977 (4)	0.6373 (3)	0.0907 (14)	
H7A	0.4509	−0.1370	0.6396	0.109*	
H7B	0.4521	−0.0800	0.6026	0.109*	
C8	0.4565 (5)	−0.0675 (5)	0.6845 (4)	0.134 (2)	
H8A	0.4546	−0.0260	0.6763	0.161*	
H8B	0.4972	−0.0776	0.6903	0.161*	
C9	0.3442 (3)	−0.1124 (4)	0.6830 (3)	0.0907 (14)	
H9A	0.3032	−0.1036	0.6778	0.109*	
H9B	0.3477	−0.1531	0.6939	0.109*	
C10	0.3674 (4)	−0.0770 (6)	0.7282 (4)	0.134 (2)	
H10A	0.3569	−0.0368	0.7201	0.161*	
H10B	0.3484	−0.0878	0.7644	0.161*	
C12	0.2463 (4)	−0.2556 (2)	0.5500 (5)	0.163 (3)	
H12A	0.2280	−0.2535	0.5880	0.196*	
H12B	0.2171	−0.2673	0.5218	0.196*	
C11	0.2713 (3)	−0.1964 (2)	0.5334 (5)	0.126 (2)	
H11A	0.2807	−0.1969	0.4922	0.151*	
H11B	0.2414	−0.1671	0.5393	0.151*	
C13	0.3666 (4)	−0.2253 (3)	0.5699 (5)	0.126 (2)	
H13A	0.3873	−0.2288	0.5332	0.151*	
H13B	0.3946	−0.2145	0.5997	0.151*	
C14	0.3422 (5)	−0.2777 (4)	0.5838 (6)	0.163 (3)	
H14A	0.3308	−0.2761	0.6245	0.196*	
H14B	0.3723	−0.3072	0.5805	0.196*	

### Atomic displacement parameters (Å^2^)

**Table:**

	*U*^11^	*U*^22^	*U*^33^	*U*^12^	*U*^13^	*U*^23^
Nd1	0.02596 (19)	0.04536 (15)	0.04536 (15)	0.000	0.000	0.000
Na1	0.0368 (17)	0.078 (3)	0.063 (2)	0.000	0.000	0.000
P1	0.0465 (8)	0.0611 (9)	0.0663 (10)	0.0090 (8)	−0.0157 (8)	−0.0007 (8)
S1	0.0324 (6)	0.0678 (9)	0.0624 (9)	0.0034 (7)	−0.0001 (7)	−0.0180 (8)
C1	0.049 (3)	0.065 (4)	0.055 (3)	0.001 (3)	0.016 (3)	−0.010 (3)
N1	0.054 (3)	0.113 (4)	0.069 (4)	0.041 (3)	−0.004 (3)	−0.008 (3)
N2	0.052 (2)	0.154 (4)	0.066 (2)	−0.005 (3)	−0.0010 (19)	0.018 (3)
N3	0.091 (3)	0.067 (3)	0.219 (6)	0.007 (2)	−0.064 (3)	0.010 (3)
O2	0.0341 (17)	0.059 (2)	0.056 (2)	−0.0013 (18)	0.0033 (18)	−0.009 (2)
O3	0.0338 (19)	0.085 (3)	0.097 (3)	0.001 (2)	0.007 (2)	−0.027 (3)
O1	0.0365 (18)	0.060 (2)	0.061 (2)	0.0045 (18)	0.0008 (18)	0.017 (2)
O4	0.119 (4)	0.212 (5)	0.071 (3)	−0.029 (4)	−0.014 (3)	0.000 (3)
O5	0.194 (6)	0.080 (3)	0.216 (6)	−0.016 (3)	−0.067 (5)	0.034 (4)
C2	0.073 (4)	0.067 (4)	0.090 (5)	0.009 (4)	0.023 (4)	−0.002 (4)
C3	0.143 (7)	0.073 (5)	0.106 (6)	0.021 (5)	0.074 (5)	−0.001 (4)
C4	0.159 (9)	0.077 (5)	0.100 (6)	−0.018 (6)	0.034 (6)	−0.031 (5)
C5	0.137 (8)	0.106 (6)	0.091 (6)	−0.034 (6)	0.003 (6)	−0.041 (5)
C6	0.086 (5)	0.082 (5)	0.070 (4)	−0.009 (4)	−0.007 (4)	−0.009 (4)
C7	0.052 (2)	0.154 (4)	0.066 (2)	−0.005 (3)	−0.0010 (19)	0.018 (3)
C8	0.119 (4)	0.212 (5)	0.071 (3)	−0.029 (4)	−0.014 (3)	0.000 (3)
C9	0.052 (2)	0.154 (4)	0.066 (2)	−0.005 (3)	−0.0010 (19)	0.018 (3)
C10	0.119 (4)	0.212 (5)	0.071 (3)	−0.029 (4)	−0.014 (3)	0.000 (3)
C12	0.194 (6)	0.080 (3)	0.216 (6)	−0.016 (3)	−0.067 (5)	0.034 (4)
C11	0.091 (3)	0.067 (3)	0.219 (6)	0.007 (2)	−0.064 (3)	0.010 (3)
C13	0.091 (3)	0.067 (3)	0.219 (6)	0.007 (2)	−0.064 (3)	0.010 (3)
C14	0.194 (6)	0.080 (3)	0.216 (6)	−0.016 (3)	−0.067 (5)	0.034 (4)

### Geometric parameters (Å, °)

**Table:**

Nd1—O1^i^	2.376 (4)	O5—C12	1.429 (10)
Nd1—O1^ii^	2.376 (4)	O5—C14	1.434 (13)
Nd1—O1	2.376 (4)	C2—C3	1.413 (10)
Nd1—O1^iii^	2.376 (4)	C2—H2A	0.9300
Nd1—O2^ii^	2.532 (4)	C3—C4	1.345 (13)
Nd1—O2	2.532 (4)	C3—H3A	0.9300
Nd1—O2^iii^	2.532 (4)	C4—C5	1.302 (13)
Nd1—O2^i^	2.532 (4)	C4—H4A	0.9300
Na1—O3	2.282 (4)	C5—C6	1.400 (11)
Na1—O3^iv^	2.282 (4)	C5—H5A	0.9300
Na1—O3^v^	2.282 (4)	C6—H6A	0.9300
Na1—O3^ii^	2.282 (4)	C7—C8	1.374 (12)
Na1—S1	3.2926 (16)	C7—H7A	0.9700
Na1—S1^v^	3.2926 (16)	C7—H7B	0.9700
Na1—S1^iv^	3.2926 (16)	C8—H8A	0.9700
Na1—S1^ii^	3.2926 (16)	C8—H8B	0.9700
P1—O1	1.499 (4)	C9—C10	1.421 (13)
P1—N1	1.613 (6)	C9—H9A	0.9700
P1—N3	1.619 (7)	C9—H9B	0.9700
P1—N2	1.637 (7)	C10—H10A	0.9700
S1—O3	1.444 (4)	C10—H10B	0.9700
S1—O2	1.453 (4)	C12—C11	1.524 (5)
S1—N1	1.513 (6)	C12—H12A	0.9700
S1—C1	1.787 (6)	C12—H12B	0.9700
C1—C6	1.365 (10)	C11—H11A	0.9700
C1—C2	1.380 (9)	C11—H11B	0.9700
N2—C7	1.400 (9)	C13—C14	1.364 (12)
N2—C9	1.465 (9)	C13—H13A	0.9700
N3—C11	1.425 (11)	C13—H13B	0.9700
N3—C13	1.478 (10)	C14—H14A	0.9700
O4—C10	1.340 (11)	C14—H14B	0.9700
O4—C8	1.424 (10)		
			
O1^i^—Nd1—O1^ii^	97.89 (6)	S1—N1—P1	127.6 (3)
O1^i^—Nd1—O1	97.89 (6)	C7—N2—C9	111.4 (6)
O1^ii^—Nd1—O1	136.50 (17)	C7—N2—P1	126.4 (5)
O1^i^—Nd1—O1^iii^	136.50 (17)	C9—N2—P1	120.7 (5)
O1^ii^—Nd1—O1^iii^	97.89 (6)	C11—N3—C13	113.8 (7)
O1—Nd1—O1^iii^	97.89 (6)	C11—N3—P1	120.7 (5)
O1^i^—Nd1—O2^ii^	151.18 (12)	C13—N3—P1	119.0 (6)
O1^ii^—Nd1—O2^ii^	72.42 (12)	S1—O2—Nd1	140.7 (2)
O1—Nd1—O2^ii^	74.32 (13)	S1—O3—Na1	122.6 (3)
O1^iii^—Nd1—O2^ii^	72.30 (12)	P1—O1—Nd1	139.6 (2)
O1^i^—Nd1—O2	72.30 (12)	C10—O4—C8	111.7 (7)
O1^ii^—Nd1—O2	74.32 (13)	C12—O5—C14	112.9 (8)
O1—Nd1—O2	72.42 (12)	C1—C2—C3	115.3 (7)
O1^iii^—Nd1—O2	151.18 (12)	C1—C2—H2A	122.4
O2^ii^—Nd1—O2	78.92 (16)	C3—C2—H2A	122.4
O1^i^—Nd1—O2^iii^	74.32 (13)	C4—C3—C2	123.9 (8)
O1^ii^—Nd1—O2^iii^	151.18 (12)	C4—C3—H3A	118.1
O1—Nd1—O2^iii^	72.30 (12)	C2—C3—H3A	118.1
O1^iii^—Nd1—O2^iii^	72.42 (12)	C5—C4—C3	118.2 (9)
O2^ii^—Nd1—O2^iii^	126.59 (10)	C5—C4—H4A	120.9
O2—Nd1—O2^iii^	126.59 (10)	C3—C4—H4A	120.9
O1^i^—Nd1—O2^i^	72.42 (12)	C4—C5—C6	122.8 (9)
O1^ii^—Nd1—O2^i^	72.30 (12)	C4—C5—H5A	118.6
O1—Nd1—O2^i^	151.18 (12)	C6—C5—H5A	118.6
O1^iii^—Nd1—O2^i^	74.32 (13)	C1—C6—C5	118.3 (8)
O2^ii^—Nd1—O2^i^	126.59 (10)	C1—C6—H6A	120.8
O2—Nd1—O2^i^	126.59 (10)	C5—C6—H6A	120.8
O2^iii^—Nd1—O2^i^	78.92 (16)	C8—C7—N2	120.1 (8)
O3—Na1—O3^iv^	119.6 (2)	C8—C7—H7A	107.3
O3—Na1—O3^v^	102.5 (2)	N2—C7—H7A	107.3
O3^iv^—Na1—O3^v^	106.8 (2)	C8—C7—H7B	107.3
O3—Na1—O3^ii^	106.8 (2)	N2—C7—H7B	107.3
O3^iv^—Na1—O3^ii^	102.5 (2)	H7A—C7—H7B	106.9
O3^v^—Na1—O3^ii^	119.6 (2)	C7—C8—O4	113.0 (9)
O3—Na1—S1	21.69 (11)	C7—C8—H8A	109.0
O3^iv^—Na1—S1	112.32 (11)	O4—C8—H8A	109.0
O3^v^—Na1—S1	123.55 (12)	C7—C8—H8B	109.0
O3^ii^—Na1—S1	89.82 (10)	O4—C8—H8B	109.0
O3—Na1—S1^v^	123.55 (12)	H8A—C8—H8B	107.8
O3^iv^—Na1—S1^v^	89.82 (10)	C10—C9—N2	110.7 (7)
O3^v^—Na1—S1^v^	21.69 (11)	C10—C9—H9A	109.5
O3^ii^—Na1—S1^v^	112.32 (11)	N2—C9—H9A	109.5
S1—Na1—S1^v^	144.97 (6)	C10—C9—H9B	109.5
O3—Na1—S1^iv^	112.32 (11)	N2—C9—H9B	109.5
O3^iv^—Na1—S1^iv^	21.69 (11)	H9A—C9—H9B	108.1
O3^v^—Na1—S1^iv^	89.82 (10)	O4—C10—C9	117.0 (9)
O3^ii^—Na1—S1^iv^	123.55 (12)	O4—C10—H10A	108.1
S1—Na1—S1^iv^	113.77 (6)	C9—C10—H10A	108.1
S1^v^—Na1—S1^iv^	77.18 (5)	O4—C10—H10B	108.1
O3—Na1—S1^ii^	89.82 (10)	C9—C10—H10B	108.1
O3^iv^—Na1—S1^ii^	123.55 (12)	H10A—C10—H10B	107.3
O3^v^—Na1—S1^ii^	112.32 (11)	O5—C12—C11	108.6 (7)
O3^ii^—Na1—S1^ii^	21.69 (11)	O5—C12—H12A	110.0
S1—Na1—S1^ii^	77.18 (5)	C11—C12—H12A	110.0
S1^v^—Na1—S1^ii^	113.77 (6)	O5—C12—H12B	110.0
S1^iv^—Na1—S1^ii^	144.97 (6)	C11—C12—H12B	110.0
O1—P1—N1	117.1 (3)	H12A—C12—H12B	108.3
O1—P1—N3	106.4 (3)	N3—C11—C12	115.6 (7)
N1—P1—N3	111.2 (4)	N3—C11—H11A	108.4
O1—P1—N2	113.1 (3)	C12—C11—H11A	108.4
N1—P1—N2	103.2 (3)	N3—C11—H11B	108.4
N3—P1—N2	105.4 (4)	C12—C11—H11B	108.4
O3—S1—O2	114.0 (3)	H11A—C11—H11B	107.5
O3—S1—N1	110.8 (3)	C14—C13—N3	111.6 (8)
O2—S1—N1	114.2 (3)	C14—C13—H13A	109.3
O3—S1—C1	103.8 (3)	N3—C13—H13A	109.3
O2—S1—C1	106.2 (3)	C14—C13—H13B	109.3
N1—S1—C1	106.8 (3)	N3—C13—H13B	109.3
O3—S1—Na1	35.73 (19)	H13A—C13—H13B	108.0
O2—S1—Na1	114.25 (17)	C13—C14—O5	118.5 (10)
N1—S1—Na1	79.9 (2)	C13—C14—H14A	107.7
C1—S1—Na1	131.7 (2)	O5—C14—H14A	107.7
C6—C1—C2	121.4 (6)	C13—C14—H14B	107.7
C6—C1—S1	121.0 (5)	O5—C14—H14B	107.7
C2—C1—S1	117.5 (5)	H14A—C14—H14B	107.1
			
O3^iv^—Na1—S1—O3	−114.4 (4)	O3—S1—O2—Nd1	−121.0 (4)
O3^v^—Na1—S1—O3	15.9 (4)	N1—S1—O2—Nd1	7.8 (5)
O3^ii^—Na1—S1—O3	142.1 (2)	C1—S1—O2—Nd1	125.3 (4)
S1^v^—Na1—S1—O3	11.0 (3)	Na1—S1—O2—Nd1	−81.7 (3)
S1^iv^—Na1—S1—O3	−90.9 (3)	O1^i^—Nd1—O2—S1	−126.9 (4)
S1^ii^—Na1—S1—O3	124.3 (3)	O1^ii^—Nd1—O2—S1	129.2 (4)
O3—Na1—S1—O2	−98.0 (4)	O1—Nd1—O2—S1	−22.3 (3)
O3^iv^—Na1—S1—O2	147.6 (2)	O1^iii^—Nd1—O2—S1	51.6 (5)
O3^v^—Na1—S1—O2	−82.1 (2)	O2^ii^—Nd1—O2—S1	54.6 (3)
O3^ii^—Na1—S1—O2	44.1 (2)	O2^iii^—Nd1—O2—S1	−73.1 (3)
S1^v^—Na1—S1—O2	−86.97 (18)	O2^i^—Nd1—O2—S1	−177.7 (3)
S1^iv^—Na1—S1—O2	171.15 (19)	O2—S1—O3—Na1	98.6 (3)
S1^ii^—Na1—S1—O2	26.33 (17)	N1—S1—O3—Na1	−31.9 (4)
O3—Na1—S1—N1	149.9 (4)	C1—S1—O3—Na1	−146.2 (3)
O3^iv^—Na1—S1—N1	35.5 (2)	O3^iv^—Na1—O3—S1	75.7 (3)
O3^v^—Na1—S1—N1	165.7 (2)	O3^v^—Na1—O3—S1	−166.5 (4)
O3^ii^—Na1—S1—N1	−68.0 (2)	O3^ii^—Na1—O3—S1	−39.9 (2)
S1^v^—Na1—S1—N1	160.9 (2)	S1^v^—Na1—O3—S1	−172.4 (2)
S1^iv^—Na1—S1—N1	59.0 (2)	S1^iv^—Na1—O3—S1	98.4 (3)
S1^ii^—Na1—S1—N1	−85.8 (2)	S1^ii^—Na1—O3—S1	−53.7 (3)
O3—Na1—S1—C1	46.3 (4)	N1—P1—O1—Nd1	−1.5 (5)
O3^iv^—Na1—S1—C1	−68.1 (3)	N3—P1—O1—Nd1	−126.5 (5)
O3^v^—Na1—S1—C1	62.2 (3)	N2—P1—O1—Nd1	118.3 (4)
O3^ii^—Na1—S1—C1	−171.6 (3)	O1^i^—Nd1—O1—P1	86.0 (3)
S1^v^—Na1—S1—C1	57.3 (3)	O1^ii^—Nd1—O1—P1	−24.3 (3)
S1^iv^—Na1—S1—C1	−44.6 (3)	O1^iii^—Nd1—O1—P1	−134.7 (4)
S1^ii^—Na1—S1—C1	170.6 (3)	O2^ii^—Nd1—O1—P1	−65.6 (4)
O3—S1—C1—C6	−131.8 (6)	O2—Nd1—O1—P1	17.5 (4)
O2—S1—C1—C6	−11.2 (6)	O2^iii^—Nd1—O1—P1	156.7 (4)
N1—S1—C1—C6	111.1 (6)	O2^i^—Nd1—O1—P1	153.6 (3)
Na1—S1—C1—C6	−157.6 (4)	C6—C1—C2—C3	3.4 (10)
O3—S1—C1—C2	53.1 (6)	S1—C1—C2—C3	178.5 (5)
O2—S1—C1—C2	173.7 (5)	C1—C2—C3—C4	−1.8 (12)
N1—S1—C1—C2	−64.0 (6)	C2—C3—C4—C5	−1.3 (14)
Na1—S1—C1—C2	27.4 (7)	C3—C4—C5—C6	2.9 (14)
O3—S1—N1—P1	154.8 (4)	C2—C1—C6—C5	−2.0 (11)
O2—S1—N1—P1	24.4 (6)	S1—C1—C6—C5	−176.9 (6)
C1—S1—N1—P1	−92.7 (5)	C4—C5—C6—C1	−1.4 (13)
Na1—S1—N1—P1	136.6 (5)	C9—N2—C7—C8	−40.8 (12)
O1—P1—N1—S1	−28.1 (6)	P1—N2—C7—C8	153.3 (8)
N3—P1—N1—S1	94.4 (6)	N2—C7—C8—O4	42.8 (13)
N2—P1—N1—S1	−153.0 (5)	C10—O4—C8—C7	−46.7 (13)
O1—P1—N2—C7	−139.0 (7)	C7—N2—C9—C10	41.8 (11)
N1—P1—N2—C7	−11.5 (8)	P1—N2—C9—C10	−151.4 (7)
N3—P1—N2—C7	105.2 (8)	C8—O4—C10—C9	54.2 (13)
O1—P1—N2—C9	56.4 (8)	N2—C9—C10—O4	−52.2 (12)
N1—P1—N2—C9	−176.2 (7)	C14—O5—C12—C11	49.2 (12)
N3—P1—N2—C9	−59.4 (8)	C13—N3—C11—C12	45.9 (12)
O1—P1—N3—C11	29.9 (9)	P1—N3—C11—C12	−162.7 (7)
N1—P1—N3—C11	−98.7 (8)	O5—C12—C11—N3	−48.4 (12)
N2—P1—N3—C11	150.2 (8)	C11—N3—C13—C14	−44.0 (14)
O1—P1—N3—C13	179.8 (8)	P1—N3—C13—C14	164.1 (9)
N1—P1—N3—C13	51.3 (9)	N3—C13—C14—O5	48.7 (16)
N2—P1—N3—C13	−59.9 (9)	C12—O5—C14—C13	−54.3 (15)

Symmetry codes: (i) −*x*+1/2, −*z*+1/2, *y*+1/2; (ii) *x*, −*y*, −*z*+1; (iii) −*x*+1/2, *z*−1/2, −*y*+1/2; (iv) −*x*+1, *y*, −*z*+1; (v) −*x*+1, −*y*, *z*.
